# Artificial intelligence transforming healthcare research: opportunities, risks, and responsible use

**DOI:** 10.36416/1806-3756/e20250433

**Published:** 2025-11-19

**Authors:** Juan C Calderon, Karla Robles-Velasco, Juliana C Ferreira

**Affiliations:** 1. Methods in Epidemiologic, Clinical, and Operations Research-MECOR-program, American Thoracic Society/Asociación Latinoamericana del Tórax, Montevideo, Uruguay.; 2. Universidad Espíritu Santo, Samborondón, Ecuador.; 3. Respiralab Research Group, Guayaquil, Ecuador.; 4. Divisão de Pneumologia, Instituto do Coração, Hospital das Clínicas, Faculdade de Medicina, Universidade de São Paulo, São Paulo (SP) Brasil.

## PRACTICAL SCENARIO

A team of early career researchers is developing a proposal for a clinical trial testing a new treatment for interstitial lung disease. They must write the introduction section and describe the randomization process, but the deadline for submitting is midnight of that day. With little time and uncertain about what to write, they turned to an artificial intelligence (AI) tool for assistance. The output appeared polished, accurate, and trustworthy, so they pasted it directly into their proposal and submitted it. However, during peer review, it was found that some references were nonexistent, and the description of randomization was incorrect and unsuitable for the trial design. What began as a minor shortcut has now compromised the validity of the proposal and the probability of getting funding.

## OPPORTUNITIES OF AI IN RESEARCH

AI is a field of computer science that develops systems capable of learning, reasoning, understanding human language, perceiving, and making decisions in ways that mimic human cognition. Common applications include voice assistants, chatbots, and large language models (LLMs), such as ChatGPT, Gemini, and DeepSeek, which are trained in massive datasets that can understand, process, and generate human-like language. 

**Figure 1:**
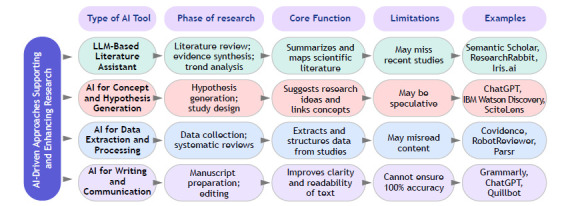
Artificial intelligence (AI)-driven tools supporting different phases of the research process. LLM: large language model.

AI has been rapidly integrated across multiple stages of the scientific process, through natural language processing (NLP) and machine learning. Specialized tools (open or subscription-based) assist in searching, summarizing, and extracting data from published literature. Examples include Elicit, Consensus, among others (Figure 1). These platforms are useful to identify relevant studies quickly, explore evidence, and address clinical or scientific questions in an efficient and evidence-informed manner. 

Beyond literature review, AI offers additional capabilities. It can process and analyze large study datasets, generate code for statistical platforms, such as R or Python, and identify patterns or associations that might otherwise be overlooked. LLMs can be used to rewrite text, proving editorial support. At the publication stage, journals are increasingly adopting AI-based systems for research integrity checks and automatically screen manuscripts for potential plagiarism or fabricated information with high accuracy.

## RISKS AND LIMITATIONS

Despite its promising landscape, there are many concerns. One of the most relevant is “hallucination,” whereby LLMs generate plausible but inaccurate or fabricated information. In our fictitious clinical scenario, the AI generated an eloquent introduction but included inexistent references. Hallucination rates for ChatGPT have been reported between 10-40%, depending on the model version.^1^ Overreliance on AI, therefore, may lead to errors that undermine the rigor and validity of research. Also, AI reports may be constructed in studies with biases, presenting incorrect answers to clinic or research questions based on disparities according to sex, race, or ethnic origin of patients.^1^

Another risk is the potential for AI to accelerate a phase of science, in which we may “produce more but understand less,” prioritizing efficiency over comprehension and making science less innovative and more vulnerable to error and bias.^2^ In academic research, plausibility and the explanatory pathway are mandatory for scientific reasoning. Ethical considerations also extend to issues of bias, reproducibility, and accountability. Recognizing these risks does not diminish the utility of AI, but rather underscores the need for cautious, transparent, and informed use.

As a result, ethical and transparent use of AI in research reporting is now emphasized by leading editorial bodies. The International Committee of Medical Journal Editors (ICMJE) recommends that authors disclose any use of AI in writing, data analysis, or figure generation, typically in the acknowledgments section.^3^ Importantly, chatbots or LLMs cannot be credited as authors, as they lack accountability for accuracy, integrity, and originality, which are essential criteria for authorship.

AI has already transformed research by streamlining literature searches, supporting data analysis, enhancing quality control, and improving manuscript preparation, particularly for non-native English speakers. However, AI should be regarded as a support tool rather than a substitute for scientific judgment or a co-author. Final decisions and interpretations must always rest with the researcher. By combining AI’s capabilities with human expertise and adhering to ethical guidelines, investigators can harness its strengths while safeguarding research integrity and reproducibility.
